# Genome Sequence of Bifidobacterium breve INIA P734 (CECT 8178), a Strain Isolated from Human Breast Milk

**DOI:** 10.1128/MRA.00871-20

**Published:** 2021-01-07

**Authors:** Lidia Rodrigo-Torres, Eva Rodríguez, Ángela Peirotén, Susana Langa, Margarita Medina, David R. Arahal, Rosa Aznar, Juan L. Arqués

**Affiliations:** a Departamento de Microbiología y Ecología, Universitat de València, Burjassot, Spain; b Colección Española de Cultivos Tipo, Universitat de València, Paterna, Spain; c Departamento Tecnología de Alimentos, INIA, Madrid, Spain; University of Maryland School of Medicine

## Abstract

The draft genome sequence of Bifidobacterium breve INIA P734, a strain shared by mother and child, is reported. It consists of 50 contigs, with 2,391,925 bp, 2,099 genes, and a G+C content of 58.8%. The genome analysis revealed the absence of antibiotic resistance and pathogenicity-related genes.

## ANNOUNCEMENT

Isolation of bifidobacteria and lactobacilli shared by mother and child as a source of probiotics has raised considerable interest in the last years (1, 2). Bifidobacterium breve is one of the bacteria most frequently isolated from human milk and the infant gut, particularly in breastfed infants (3–5).

Breast milk and infant fecal samples were plated on reinforced clostridial medium (RCM) agar with 0.3 g/liter of methyl blue and incubated anaerobically for 72 h at 37°C for differential isolation of bifidobacteria. Isolates were identified by 16S rRNA gene sequence comparison, and discrimination of bifidobacterial strains within mother-child pairs was established by pulsed-field gel electrophoresis (PFGE) typing (2). *B. breve* INIA P734 (CECT 8178) was isolated from human milk and corresponds to a breast milk-infant feces pair with interesting probiotic (2) and technological (6) properties. *B. breve* is among the species listed as safe by the European Food Safety Authority (EFSA) for the Qualified Presumption of Safety (QPS) status, and that could underwrite its industrial applications for food supplements or fermented products. Therefore, in order to get a deeper knowledge of the probiotic potential and safety properties of this strain, whole-genome sequencing was undertaken. Culturing for genomic DNA (gDNA) isolation, gDNA extraction, and library preparation was performed by GenProbio srl (Parma, Italy), following the methodology described by Lugli et al. (7). Briefly, cells were inoculated in de Man-Rogosa-Sharpe (MRS) medium (Scharlau Chemie) supplemented with 0.05% (wt/vol) l-cysteine hydrochloride and incubated at 37°C in an anaerobic atmosphere. Cells from 10 ml of an overnight culture were harvested by centrifugation at 6,000 rpm for 8 min, and the obtained cell pellet was used for DNA extraction using the GenElute bacterial genomic DNA kit (Sigma-Aldrich) following the manufacturer’s guidelines. A genome library was generated using the TruSeq Nano DNA kit. The genome sequences were retrieved using an Illumina MiSeq platform with 2 × 250-bp paired-end reads. A total of 2 × 734,895 raw reads comprising 368,000,944 bp were obtained. Reads, quality controlled with FastQC, were assembled with the software MIRA v4.0.2 (8), and evaluation of the final assembly was done with QUAST v4.3 (9) and CheckM v1.0.7 (10) (with the options lineage_wf and reduced_tree) prior to annotation with Prokka v1.12 (11) (with the options compliant, addgenes, and rfam) and Rapid Annotation using Subsystems Technology (RAST) v2.0 (12) (with the parameters Genetic code 11, Annotation scheme “RASTtk,” and the option of Automatically fix errors). The genome was screened with the following tools recommended by EFSA (13) to investigate the relevant genes involved in food safety: (i) Resistance Gene Identifier (RGI) of the Comprehensive Antibiotic Resistance Database (CARD) (14) (default parameters) to predict the genes involved in antimicrobial resistance; (ii) PathogenFinder (15) to study bacterial pathogenicity, selecting “All” as the model and “Assembled/Genome Contigs*” as the sequencing platform; and (iii) the BAGEL4 Web server (16) (default parameters) to predict the genes involved in the synthesis of bacteriocins and ribosomically synthetized and posttranslationally modified peptides (RiPPs). The draft achieved comprises 50 contigs with 2,391,925 bp and a G+C content of 58.8%. The largest contig is 241,418 bp, and the *N*_50_ value is 217,975 bp, with a completeness of 100% and a contamination level of 0.42% (according to the CheckM results). The annotation yielded a total of 2,099 genes (2,031 proteins, 55 tRNAs, 2 rRNAs, and 11 other RNAs). Antibiotic resistance genes and pathogenic and bacteriocin-encoding genes were not found with the above-mentioned tools at the time of analysis, and accordingly, no antimicrobial resistance was detected phenotypically based on the procedures and cutoff values of the EFSA guidance (13).

### Data availability.

The genome sequence and annotation of Bifidobacterium breve INIA P734 have been deposited in the ENA under accession no. CABFNK01000000 (study accession no. PRJEB32824, BioSample accession no. SAMEA5675151, experiment accession no. ERX4268605, and run accession no. ERR4321747). The version described in this paper is the first version.
